# Icariin Augments Bone Formation and Reverses the Phenotypes of Osteoprotegerin-Deficient Mice through the Activation of Wnt/****β****-Catenin-BMP Signaling

**DOI:** 10.1155/2013/652317

**Published:** 2013-11-04

**Authors:** Xiao-Feng Li, Hao Xu, Yong-Jian Zhao, De-Zhi Tang, Guo-Hua Xu, Jonathan Holz, Jing Wang, Shao-Dan Cheng, Qi Shi, Yong-Jun Wang

**Affiliations:** ^1^Spine Research Institute, Shanghai University of Traditional Chinese Medicine, Shanghai 200032, China; ^2^Longhua Hospital, Shanghai University of Traditional Chinese Medicine, Shanghai 200032, China; ^3^Shanghai Changzheng Hospital, Shanghai 200003, China; ^4^Biology Department, St. John Fisher College, Rochester, NY 14618, USA

## Abstract

Icariin has been mostly reported to enhance bone fracture healing and treat postmenopausal osteoporosis in ovariectomized animal model. As another novel animal model of osteoporosis, there is few publication about the effect of Icariin on osteoprotegerin-deficient mice. Therefore, the goal of this study is to find the effect on bone formation and underlying mechanisms of Icariin in osteoprotegerin (OPG) knockout (KO) mice. We found that Icariin significantly stimulated new bone formation after local injection over the surface of calvaria at the dose of 5 mg/kg per day. With this dose, Icariin was also capable of significantly reversing OPG-deficient-induced bone loss and bone strength reduction. Real-time PCR analysis showed that Icariin significantly upregulated the expression of BMP2, BMP4, RUNX2, OC, Wnt1, and Wnt3a in OPG KO mice. Icariin also significantly increased the expression of AXIN2, DKK1, TCF1, and LEF1, which are the direct target genes of **β**-catenin signaling. The in vitro studies showed that Icariin induced osteoblast differentiation through the activation of Wnt/**β**-catenin-BMP signaling by in vitro deletion of the **β**-catenin gene using **β**-catenin^fx/fx^ mice. Together, our findings demonstrate that Icariin significantly reverses the phenotypes of OPG-deficient mice through the activation of Wnt/**β**-catenin-BMP signaling.

## 1. Introduction

Osteoporosis remains a major threat to public health. Approximately 40% of women and 13% of men aged 50 or older will experience at least one fracture during the remainder of their lifetime. The risk of osteoporotic fracture increases with age, prior fractures, smoking, and systemic corticosteroid use [1]. Aging-associated bone loss can be divided into two phases: a rapid phase starting at menopause and a slow phase occurring about 8 years after menopause and persisting until death. The rapid phase is caused by estrogen insufficiency and mainly affects trabecular bone. In contrast, the slow phase is a process believed to be the result of a more complex and less well-understood etiology causing loss of both trabecular and cortical bone and affecting both men and women [2].

Current treatment regiments for osteoporosis fall into two categories. Antiresorptive drugs such as estrogen and estrogen-receptor analogues inhibit osteoclast function, while bisphosphonates and anabolic drugs induce bone formation. Although estrogen replacement has been shown to improve BMD and reduce incidence of fracture during early menopause, its prolonged use is restricted because of potential complications such as breast cancer, uterine bleeding, and cardiovascular events. The incidence of osteonecrosis of the jaw seems relatively low in patients receiving oral bisphosphates for osteoporosis or Paget's disease and considerably higher in patients with malignancy receiving high doses of intravenous bisphosphates [3, 4]. Despite an excellent safety profile for parathyroid hormone (PTH), concerns arise from its persistence after discontinuation without sequential use of anti-resorptive drugs [5]. In addition, PTH is contraindicated for patients at risk for osteosarcoma. The belief that combined use of both drug types may have a synergistic effect on BMD is not fully supported by some observational studies [6]. These potential limitations of existing drugs are sufficient to warrant development of novel therapies.

Chinese herbal medicine has been widely used for thousands of years for the treatment of bone diseases. Epimedium pubescens is one of the most frequently used herbs in compounds that are prescribed for the treatment of osteoporosis. In postmenopausal women, the flavonoids in epimedium pubescens prevent bone loss [7]. Icariin, (C_33_H_40_O_15_; molecular weight: 676.67) is a flavonol, a class of flavonoid, and can be derived from several species of plants in the Epimedium family commonly known as Horny Goat Weed or Yin Yang Huo. This compound has been reported to be a potent enhancer of bone fracture healing [8], and extracts containing it can reduce the occurrence of postmenopausal osteoporosis in ovariectomized rats [9–11].

Osteoporosis results from an imbalance between bone resorption and bone formation, whereas healthy bone requires balanced interactions between osteoblasts and osteoclasts [12]. The dynamic balance between these two cell types is necessary for bone remodeling. Increased osteoclast activity induces erosion of trabecular bone and fragile bones. Conversely, increased osteoblast activity increases bone density, which is associated with bone deformity and osteopetrosis [12]. Osteoprotegerin (OPG), a naturally occurring inhibitor of the receptor activator of NF-*κ*B ligand [13], binds RANKL with high affinity, preventing RANKL from interacting with RANK on osteoclasts. This binding negatively regulates osteoclast formation and thus prevents osteoporosis. Imbalance between RANKL and OPG, resultant to estrogen shortage, glucocorticoid application, or immunomediated disorganizations induces osteoclastogenesis and accelerates bone resorption [14]. OPG-deficient (OPG^−/−^) mice develop severe osteoporosis and exhibit a remarkable increase in osteoclast number as adults [14]. OPG-deficient (OPG) mice exhibited severe osteoporosis due to enhanced osteoclastogenesis as adults [15, 16]. Deficiency of OPG in mice induces osteoporosis caused dominantly by enhanced bone resorption but also accelerates bone formation [17]. OPG^−/−^ mice are also a useful model for screening therapeutic agents against bone loss [18]. Although it has been shown that Icariin improves BMD and bone strength in ovariectomized rats in vivo [9–11] and that Icariin promotes proliferation, differentiation and activity of osteoblastic cells in vitro [19–21]. As a novel animal model of osteoporosis, there is less publication about the effect of Icariin on OPG-deficient mice. Until now, only one paper reported that the beneficial effects of Icariin on bone are diminished in OPG^−/−^ mice partly, not totally [22], suggesting that Icariin may still promote bone formation in OPG^−/−^ mice. However, the underlying mechanisms of Icariin in OPG^−/−^ mice are unknown. This study aimed to determine the effect of Icariin on bone formation and the underlying mechanisms in OPG-deficient mice.

## 2. Materials and Methods

### 2.1. In Vivo Periosteal Bone Formation Assay

All surgical protocols related to the use of mice were approved by Institutional Review Board of the Shanghai University of Traditional Chinese Medicine. Four-week ICR Swiss mice were injected subcutaneously onto the calvarial surface with or without the treatment of Icariin twice a day for 5 consecutive days at the doses of 1, 3, and 5 mg/kg per day (4 mice per group) following the reported methods [23]. To measure mineral appositional rate (MAR, *μ*m/day) and bone formation rate (BFR, *μ*m^2^/*μ*m/day), double calcein labeling was performed at days 7 and 14 by intraperitoneal injection (20 mg/kg), and mice were euthanized 2 days after the second labeling. The double labeling was examined in plastic sections. The dissected calvarial samples were fixed in 75% ethanol and embedded in methyl methacrylate. Transverse sections of mouse calvaria were cut at 3 *μ*m thickness and unstained sections were viewed under a fluorescent microscope. MAR and BFR were measured using the OsteoMeasure System (OsteoMetrics, Atlanta, GA, USA) [24].

### 2.2. Osteoprotegerin Knockout Mouse Genotyping and Treatment

Twelve 3-month-old osteoprotegerin knockout (OPG^−/−^) mice and six 3-month-old osteoprotegerin wild-type (OPG^+/+^) mice in S129 background were purchased from Shanghai Research Center for Biomodeling Organisms, China. The genotype of OPG^−/−^ mice were determined by PCR. The OPG WT allele was detected using the forward primer 5′-GTAACGCCCTTCCTCACACTCACA-3′ and the reverse primer 5′-ATGGCCATTCAGCAGTAGCCTATG-3′. The OPG KO allele was detected using the forward primer 5′-GTAACGCCCTTCCTCACACTCACA-3′ and the reverse primer 5′-GTGGGGGTGGGGTGGGATTAGATA-3′. All the mice were randomized by body weight into 3 groups (*n* = 6/group): (1) OPG wild type mice with vehicle control (WT + VEH), (2) OPG knockout mice with vehicle control (KO + VEH), and (3) OPG knockout mice with Icariin (KO + ICN, 5 mg/kg/day). Vehicle or Icariin was administered by intraperitoneal injection once a day for 8 weeks. Then, mice were sacrificed and the specimens were harvested for evaluation.

### 2.3. uCT Analysis

The fourth lumbar vertebrae (L4) were dissected and fixed in 4% paraformaldehyde and scanned at a 18 *μ*m voxel size using the uCT scanner (uCT-80, Scanco Medical AG, Bassersdorf, Switzerland). The trabecular bone under the growth plate was segmented using a contouring tool, and the contours were morphed automatically to segment the trabecular bone on all slices. The 3D images were constructed and analyzed with the evaluation software of the uCT system.

### 2.4. Histological Evaluation

After uCT analysis, the fourth lumbar vertebrae were decalcified, dehydrated, rinsed with dimethylbenzene, and then embedded in olefin. Sections cut 7 *μ*m thick were obtained and stained with Orange G for routine morphologic analysis. Histomorphometric assessment was performed using an image autoanalysis system (Olympus BX50; Japan). Trabecular bone was measured in the area of the secondary spongiosa 0.5 mm apart from the growth plate (2 mm area). All values were represented as the mean of three measurements in three nonconsecutive sections. Histomorphometric parameters were calculated and expressed according to the recommendations made by the American Society for Bone and Mineral Research (ASBMR) nomenclature committee [19, 25]. The image analysis system automatically determined the measuring areas, and the structural parameters were presented as trabecular bone volume (BV/TV, %) and trabecular number (Tb. N, 1/mm).

### 2.5. Biomechanical Testing

After careful removal of soft tissues, the left femoral samples were soaked in PBS for 3 hours for thorough hydration. To determine changes in mechanical properties, femoral shafts were tested to failure via three-point bending using an Instron Dynamite 8841 servohydraulic material-testing device equipped with a 1 kN load cell. Data collection was performed with Bluehill software (Instron, Norwood, MA, USA). The anterior surface of the femoral shaft was placed on the two support beams separated by an 8 mm span. Femurs were bent to failure along the mediolateral axis at a rate of 5 mm/minute. Force and deformation data were collected and analyzed to calculate the maximal force (N), yield force (N), stiffness (N/mm), and energy (N·mm^2^) [26].

### 2.6. Total RNA Extraction and Real-Time PCR Analysis

Total RNA was isolated from body tissues (the fifth lumbar vertebrae) or cells (bone marrow stromal cells) using TRIzol reagent (Sigma, St. Louis, MO, USA), according to the manufacturer's protocol. cDNA was synthesized using the iScript cDNA synthesis kit (Bio-Rad, Hercules, CA, USA) following the manufacturer's protocol. The cDNA was then amplified by PCR (95°C for 15 min followed by 45 cycles, 95°C for 20 seconds, 58°C for 20 seconds, and 72°C for 30 seconds) with Absolute QPCR SYBR Green Master Mix (Thermo Scientific) in 20 *μ*L buffered solution containing 2 *μ*L of the diluted (1 : 5) reverse transcription product in the presence of the sense and antisense primers, the names and sequences of which were listed in Table 1. Each sample was assessed in triplicate.

### 2.7. Bone Marrow Stromal Cell Culture and Treatment

Bone marrow stromal cells were obtained from the bone marrow of the femur and bilateral tibia of C57/BL6 mice. Cells were seeded in 6-well plates at a density of 5 × 10^6^ cells per well and maintained in DMEM-HG plus 10% fetal bovine serum (FBS) and 1% penicillin-streptomycin (Invitrogen, Carlsbad, CA, USA) and were cultured with 5 mM of *β*-glycerophosphate, 100 mM of L-ascorbic acid, and 0.1 mM of dexamethasone (Sigma, St. Louis, MO, USA) in basic medium for osteoblast differentiation. Cell culture medium was changed every 3 days. After 6 days in culture, cells were treated with Icariin at doses of 0, 1, 10, and 50 *μ*M for 2 days. Total RNA was extracted for real-time PCR analysis. All reactions were performed in triplicate independently.

### 2.8. ALP Staining Assay

After cultured in 6-well plates with Icariin (0, 1, 10, and 50 *μ*M) for 2 days, the bone marrow stromal cells were fixed in 10% neutral buffered formalin for 15 min, washed, and then incubated with ALP staining buffer, NBT-BCIP (Bio-Rad, Hercules, CA, USA) at 370°C for 30 min. The reaction was performed in triplicate independently.

### 2.9. Western-Blot Analysis

Primary bone marrow stromal cells were seeded in 6-well plates at a density of 5 × 10^6^ cells per well. After 6 days in culture, cells were treated with Icariin at doses of 0, 1, 10, and 50 *μ*M for 2 days. Cell lysates were extracted with E-PER protein extraction reagents (Thermo Scientific, Waltham, MA, USA) according to the manufacturer's protocol. Proteins were transblotted onto a PVDF membrane (Bio-Rad, Hercules, CA, USA), and the membrane was blocked with 5% milk in phosphate buffered saline containing 0.1% Tween-20 (PBST) for 1 hour at room temperature. After incubation with the primary antibody overnight at 4°C and the horseradish peroxidase (HRP)—conjugated secondary antibodies (Thermo Scientific, Waltham, MA, USA) for 1 hour at room temperature, protein expression was detected using a SuperSignal West Femto Maximum Sensitivity Substrate Kit (Thermo Scientific, Waltham, MA, USA). The polyclonal rabbit anti-active-*β*-catenin antibody was obtained from Cell Signaling Technologies (Beverly, MA, USA). After the immunocomplex was removed by stripping buffer (Chemicon, Temecula, CA, USA), the same membrane was reblotted with mouse anti-*β*-actin antibody (Sigma, St. Louis, MO, USA) for the loading control.

### 2.10. Dual Luciferase Assay

Primary bone marrow stromal cells were seeded at a density of 5 × 10^6^ cells per well in a 96-well plate. The *β*-catenin signaling reporter construct, TOPGAL, and the SV40-Renilla luciferase construct were cotransfected into the cells using FuGENE HD Transfection Reagent (Invitrogen, Carlsbad, CA, USA). Cells were then treated with Icariin at doses of 0, 1, 10, and 50 *μ*M for 2 days. Cell lysates were extracted and the relative amounts of Renilla and firefly luciferase were measured using a dual luciferase assay kit (Promega, Madison, WI, USA). The Renilla/firefly luciferase ratio was calculated and normalized against the control.

### 2.11. In Vitro Deletion of the *β*-Catenin Gene

In vitro deletion of the *β*-catenin gene was performed as previously described [26, 27]. Briefly, the *β*-catenin^fx/fx^ mice were genotyped and identified by PCR using the following primers: upper primer 5′-AAGGTAGAGTGATGAAAGTTGTT-3′ and lower primer 5′-CACCATGTCCTCTGTCTATTC-3′. Primary bone marrow stromal cells isolated from the *β*-catenin^fx/fx^ mice were seeded in 6-well culture plates at a density of 5 × 10^6^ cells per well and cultured for 6 days. Cells were infected with Ad-GFP or Ad-Cre (Titer: 4 × 10^8^ pfu/mL; Baylor College of Medicine, Houston, TX, USA) for 3 days. Ad-GFP was used as a control and to monitor infection efficiency. After recovery for 2 days, cells were treated with or without Icariin (50 *μ*M) for 2 days. qPCR was performed to examine changes in the mRNA expression of BMP2, BMP4, OC, and ALP. All reactions were performed in triplicate independently.

### 2.12. Statistical Analysis

Data were expressed as mean ± SD and analyzed by SPSS 16.0 software. Statistical comparisons between results from multiple groups were analyzed using one-way ANOVA followed by Dunnett's test. For experiments involving two groups, an unpaired Student's* t-*test was performed. A value of *P* < 0.05 was considered statistically significant.

## 3. Results

### 3.1. Icariin Increased New Bone Formation

**Figure 1:**
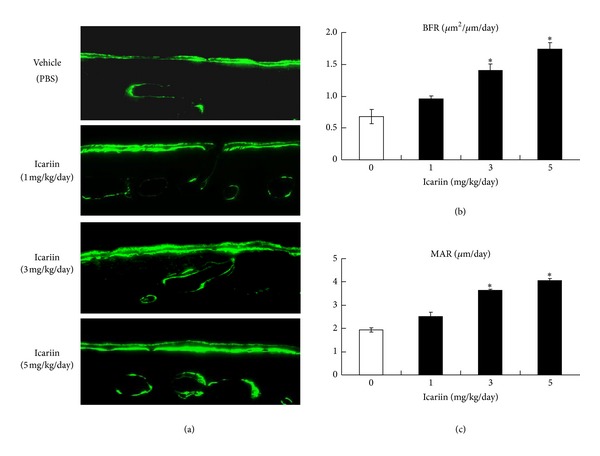
Icariin stimulated local bone formation in mouse calvaria. (a) Transverse sections of mouse calvaria were cut at 3 *μ*m thickness and unstained sections were viewed under a fluorescent microscope. The data showed that Icariin (3 mg/kg/day and 5 mg/kg/day) significantly increased new bone formation. Mineral appositional rate (MAR) (b) and bone formation rate (BFR) (c) were quantified. Data were expressed as mean ± SD, *n* = 4. Significant increases in MAR and BFR of the Icariin-treated group (3 mg/kg/day and 5 mg/kg/day) were observed. *P < 0.05, unpaired Student's *t*-test (compared with vehicle control).

To analyze whether Icariin stimulates new bone formation on the calvariae, Icariin injection (1, 3, and 5 mg/kg per day) onto mouse calvariae was followed by calcein double labeling at days 7 and 14. Calcein double labeling of undecalcified skull tissue sections showed that Icariin promoted local bone formation in a dose-dependent manner (Figure 1(a)). Furthermore, histomorphometric analysis revealed significant and dose-dependent increases in bone formation rate (BFR) (Figure 1(b)) and mineral appositional rate (MAR) (Figure 1(c)) in Icariin-treated groups, compared to the control group. The dose of 5 mg/kg per day most efficaciously stimulated bone formation.

### 3.2. Icariin Prevented Bone Loss in Osteoprotegerin-Deficient Mice

**Figure 2:**
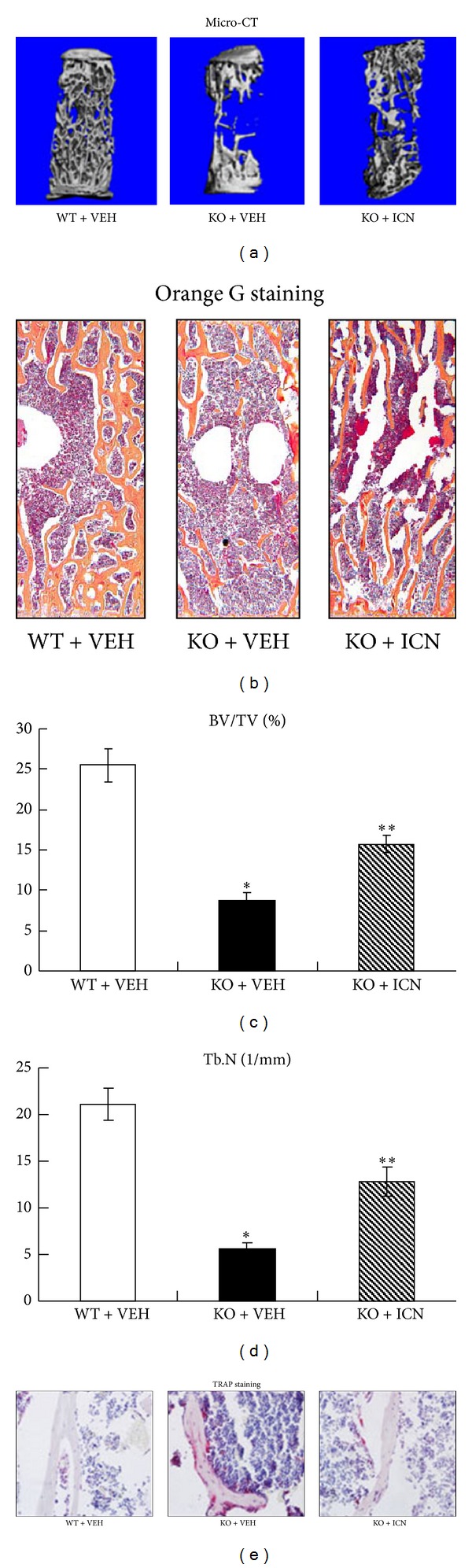
Icariin prevented bone loss in OPG-deficient mice. Fourth lumbar vertebrae (L4) samples were obtained from mice of the following groups: (1) OPG wild-type mice with vehicle control (WT + VEH), (2) OPG knockout mice with vehicle control (KO + VEH), and (3) OPG knockout mice with Icariin (KO + ICN, 5 mg/kg/day). Data were expressed as mean ± SD, *n* = 6. (a) uCT analysis data of L4 samples showed that Icariin prevented bone loss in OPG-deficient mice. (b) Histologic sections of L4 samples stained with Orange *G* were obtained and analyzed. Bone histomorphometric analysis showed that trabecular bone volume (BV/TV, %) (c) and Trabecular number (Tb. N, 1/mm) (d) were significantly increased in Icariin-treated OPG KO mice, compared to the vehicle-treated OPG KO mice. TRAP staining showed that Icariin significantly decreased osteoclast number in OPG KO mice (e). *P < 0.05, unpaired Student's *t*-test (OPG KO + VEH group versus OPG WT + VEH group) and **P < 0.05, unpaired Student's *t*-test (OPG KO + ICN group versus OPG KO + VEH group).

We next analyzed the systemic effects of Icariin at the dose of 5 mg/kg per day in OPG knockout mice. Fourth lumbar vertebrae (L4) were obtained from mice of the following groups: (1) OPG wild-type mice with vehicle control (WT + VEH), (2) OPG knockout mice with vehicle control (KO + VEH), and (3) OPG knockout mice with Icariin (KO + ICN, 5 mg/kg/day). uCT analysis (Figure 2(a)) and histological examination (Figure 2(b)) of the L4 samples demonstrated an apparent bone loss in OPG KO mice compared to OPG WT mice. Intraperitoneal injection of Icariin for 8 weeks resulted in a partial recovery of the trabecular structure in OPG KO mice. Histomorphometric analysis showed that treatment with Icariin significantly increased trabecular bone volume (Figure 2(c)) and trabecular number (Figure 2(d)) in OPG KO mice. TRAP staining showed that Icariin significantly decreased osteoclast number in OPG KO mice (Figure 2(e)). Taken together, these findings indicate that Icariin can prevent bone loss in osteoprotegerin-deficient mice.

### 3.3. Icariin Promoted Bone Strength in Osteoprotegerin-Deficient Mice

**Figure 3:**
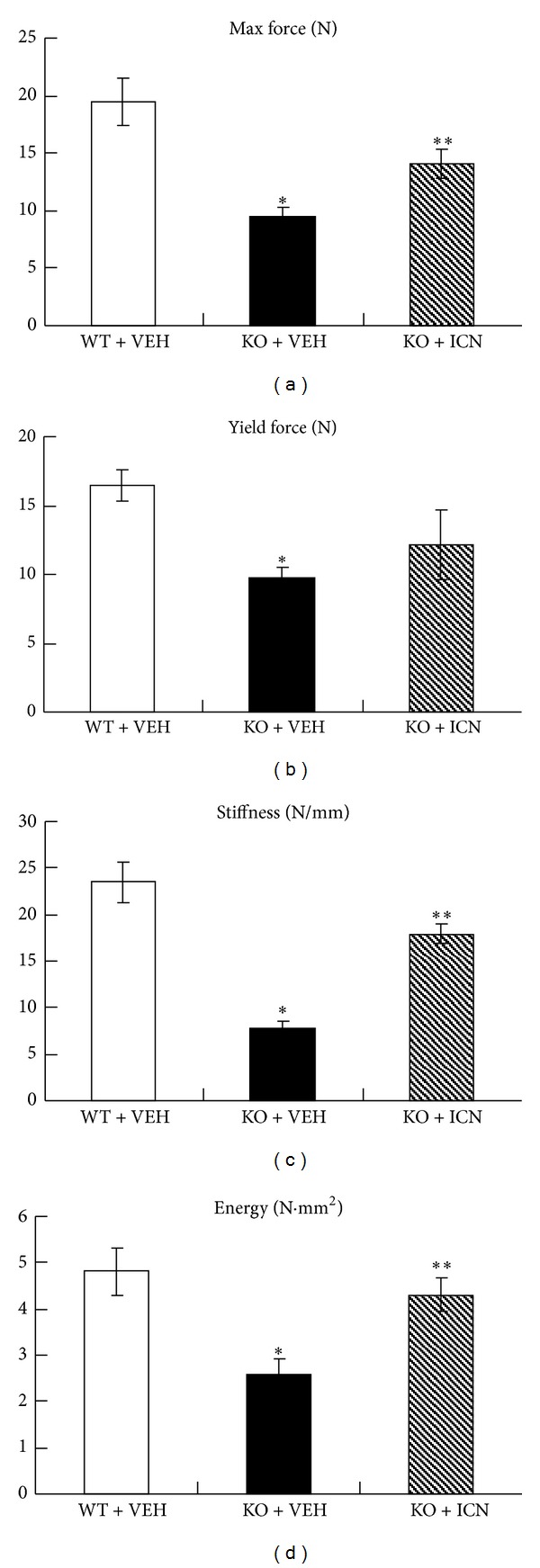
Icariin promoted bone strength in OPG-deficient mice. To further determine if treatment with Icariin improves biomechanical properties of bone, a three-point bending test was performed on femoral shaft samples obtained from three different groups. Data were expressed as mean ± SD, *n* = 6. Deletion of the OPG gene caused a dramatic reduction in maximal force (a), yield force (b), bone stiffness (c), and bone energy (d). Treatment with Icariin significantly reversed the reduced maximal force, stiffness, and energy in OPG KO mice. *P < 0.05, unpaired Student's *t*-test (OPG KO + VEH group versus OPG WT + VEH group) and **P < 0.05, unpaired Student's *t*-test (OPG KO + ICN group versus OPG KO + VEH group).

To further determine if treatment with Icariin improves the biomechanical properties of bone, a three-point bending test was performed on femoral shaft samples taken from mice in the following treatment groups: (WT + VEH), (KO + VEH), and (KO + ICN, 5 mg/kg/day). Deletion of the OPG gene caused a dramatic reduction in maximal force (Figure 3(a)), yield force (Figure 3(b)), bone stiffness (Figure 3(c)), and bone energy (Figure 3(d)). Treatment with Icariin for 8 weeks significantly reversed the reductions in maximal force (Figure 3(a)), stiffness (Figure 3(c)), and energy (Figure 3(d)) in OPG KO mice. This demonstrates the ability of Icariin to significantly promote bone mechanical strength in osteoprotegerin-deficient mice.

### 3.4. Icariin Stimulated Osteoblast Differentiation in Osteoprotegerin-Deficient Mice

**Figure 4:**
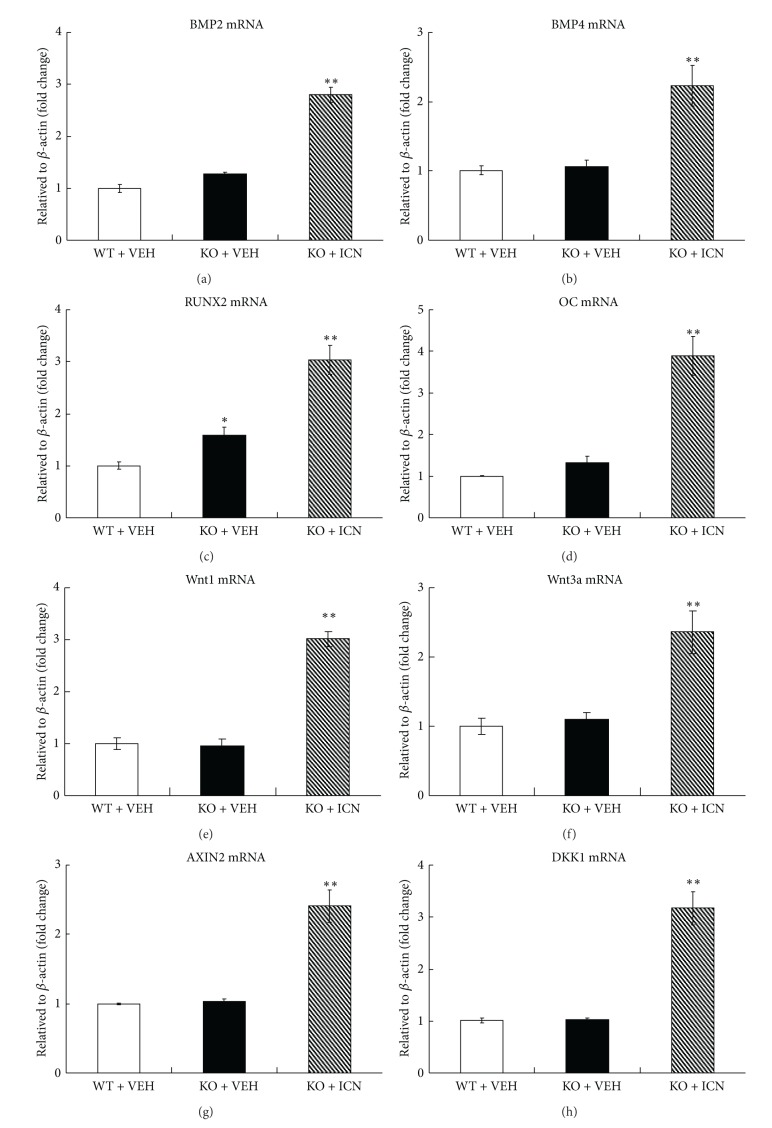

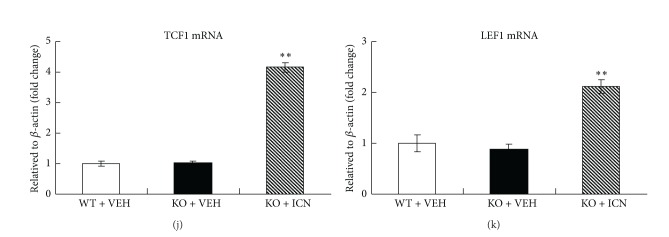
Icariin stimulated osteoblast differentiation in OPG-deficient mice. Total RNA was extracted from the fifth lumbar vertebrae (L5) and qPCR analyses were performed. Data were expressed as mean ± SD, *n* = 3. Compared to the vehicle control, Icariin significantly increased the expression of osteoblast-specific marker genes such as BMP2 (a), BMP4 (b), RUNX2 (c), and OC (d) of OPG KO mice. Compared to the vehicle control, Icariin significantly increased the expression of Wnt1 (e) and Wnt3a (f) in OPG KO mice. Icariin also significantly upregulated the expression of AXIN2 (g), DKK1 (h), TCF1 (i), and LEF1 (j), which are the direct target genes of *β*-catenin signaling. *P < 0.05, unpaired Student's *t*-test (OPG KO + VEH group versus WT + VEH group) and **P < 0.05, unpaired Student's *t*-test (OPG KO + Icariin group versus OPG KO + VEH group).

To determine possible effects of Icariin on osteoblast differentiation, total RNA was extracted from the fifth mouse lumbar vertebrae (L5) of the groups described previously and qPCR analyses were performed. Compared to vehicle control, Icariin significantly increased the expression of osteoblast-specific marker genes in OPG KO mice. The expression of the following genes were found to be increased: BMP2 (2.7-fold) (Figure 4(a)), BMP4 (2.2-fold) (Figure 4(b)), RUNX2 (3.0-fold) (Figure 4(c)), and OC (3.9-fold) (Figure 4(d)). Compared to the vehicle control, Icariin significantly increased the expression of Wnt1 (Figure 4(e)) and Wnt3a (Figure 4(f)) in OPG KO mice, with 3.0-fold and 2.4-fold changes, respectively. Icariin also significantly upregulated the expression of AXIN2 (2.4-fold) (Figure 4(g)), DKK1 (3.2-fold) (Figure 4(h)), TCF1 (4.2-fold) (Figure 4(j)), and LEF1 (2.1-fold) (Figure 4(k)), which are the direct target genes of *β*-catenin signaling. These changes suggest that Icariin may have stimulated osteoblast differentiation through activation of *β*-catenin signaling in osteoprotegerin-deficient mice.

### 3.5. Icariin Activated Canonical Wnt/*β*-Catenin Signaling In Vitro

**Figure 5:**
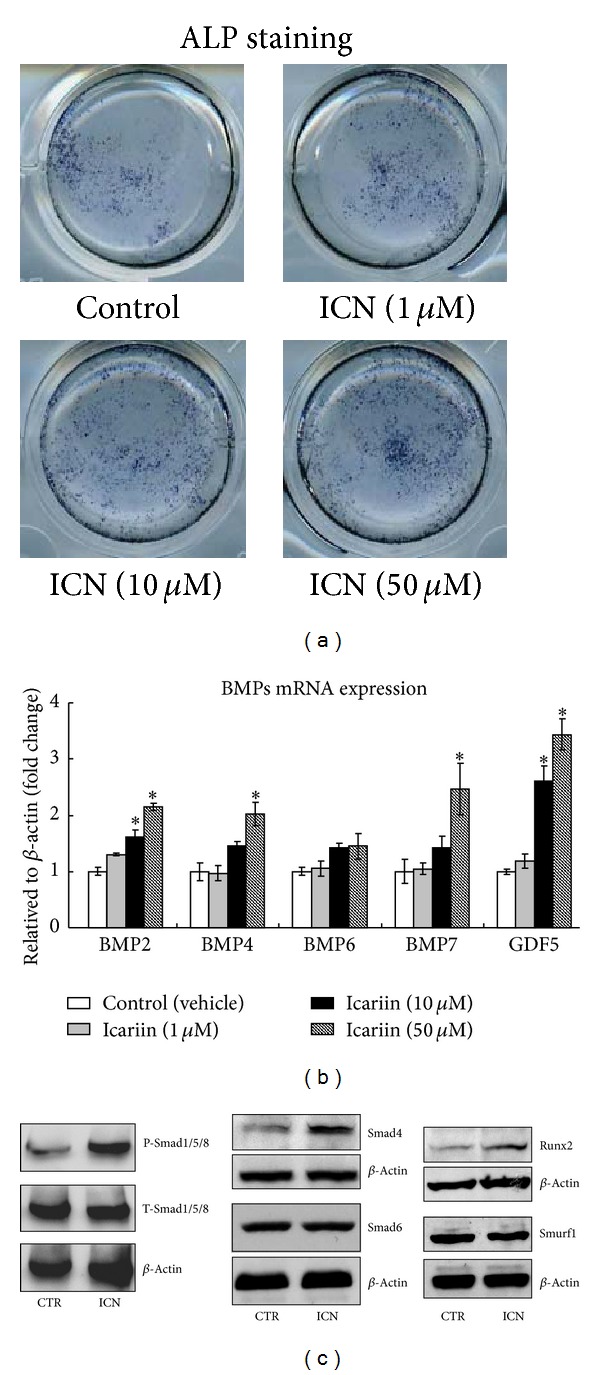
Icariin activated BMPs/Smads/Runx2 signaling in bone marrow stromal cells. Primary bone marrow stromal cells were cultured with or without Icariin (1, 10, and 50 *μ*M) for 2 days; then the ALP staining assay was performed and the mRNA expression of BMP ligands, Smads protein expression, and Runx2 protein expression were,respectively, examined. The results of ALP staining indicated that a high dose of Icariin (50 *μ*M) enhanced the expression of ALP (a). Data were expressed as mean ± SD, *n* = 3. The qPCR data (b) showed that a high dose of Icariin (50 *μ*M) significantly increased the expression of BMP2, BMP4, BMP7, GDF5, and the Western-blot analysis results (c) showed that a high dose of Icariin (50 *μ*M) increased the protein level of Phosphorylated-Smads1/5/8, Smad4, and Runx2. *P < 0.05, unpaired Student's* t-*test (Icariin versus vehicle control).

**Figure 6:**
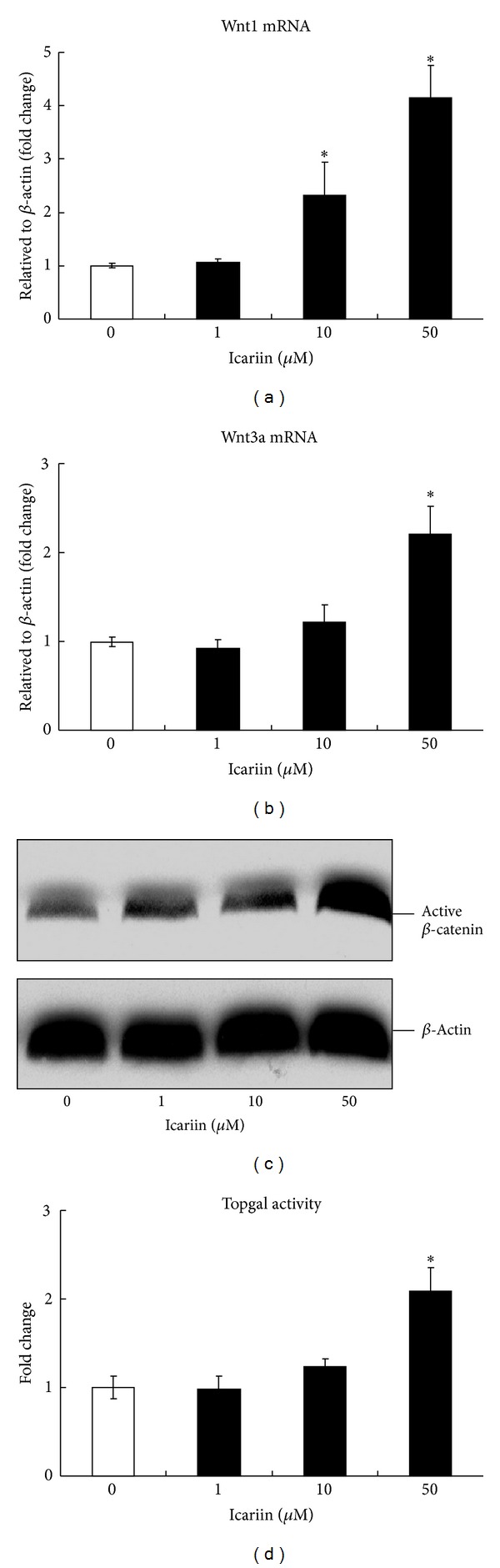
Icariin activated canonical Wnt/*β*-catenin signaling in bone marrow stromal cells. Primary bone marrow stromal cells were cultured with or without Icariin (1, 10, and 50 *μ*M) for 2 days; then the mRNA expression of Wnt ligands, *β*-catenin protein expression, and *β*-catenin signaling reporter (TOPGAL) activity were, respectively, examined. Data were expressed as mean ± SD, *n* = 3. 50 *μ*M Icariin significantly increased the mRNA expression of Wnt1 (a) and Wnt3a (b) and significantly upregulated active-*β*-catenin protein levels (c). 50 *μ*M Icariin significantly increased the luciferase activity of the TOPGAL reporter (d). *P < 0.05, unpaired Student's* t-*test (Icariin versus vehicle control).

To further determine the mechanism of Icariin-induced osteoblast differentiation, we performed in vitro study. Primary bone marrow stromal cells were cultured with or without Icariin (1, 10, and 50 *μ*M) for 2 days, then the ALP staining assay was performed, and the mRNA expressions of BMP ligands, Smads protein, Runx2 protein were, respectively, examined. The results of ALP staining showed that a high dose of Icariin (50 *μ*M) stimulated osteoblast differentiation, demonstrated by the expression of ALP, was enhanced after treatment with Icariin (Figure 5(a)). The qPCR data (Figure 5(b)) showed that a high dose of Icariin (50 *μ*M) significantly increased the expression of BMP2, BMP4, BMP7, and GDF5, and the Western-blot analysis results (Figure 5(c)) showed that a high dose of Icariin (50 *μ*M) increased the protein level of Phosphorylated-Smad1/5/8, Smad4, and Runx2. Our recent studies have demonstrated that BMP expression is activated by canonical Wnt/*β*-catenin signaling in osteoblast cells and in bone marrow stromal cells [26, 27]. To determine if Icariin affects Wnt/*β*-catenin signaling, we examined its effects on the mRNA expression of Wnt ligands, *β*-catenin protein expression, and *β*-catenin signaling reporter activity in bone marrow stromal cells. The qPCR data showed that Icariin significantly up-regulated the expression of Wnt1 (Figure 6(a)) and Wnt3a (Figure 6(b)) in a dose-dependent manner. Western-blot analysis showed that a high dose of Icariin (50 *μ*M) dramatically increased the active *β*-catenin protein level (Figure 6(c)). Consequently, a high dose of Icariin (50 *μ*M) significantly promoted TOPGAL (*β*-catenin signaling reporter) activity, eliciting a 2-fold increase (Figure 6(d)). These findings demonstrate that Icariin can stimulate canonical Wnt/*β*-catenin signaling in bone marrow stromal cells.

### 3.6. Icariin-Induced Osteoblast Differentiation Was *β*-Catenin Dependent

**Figure 7:**
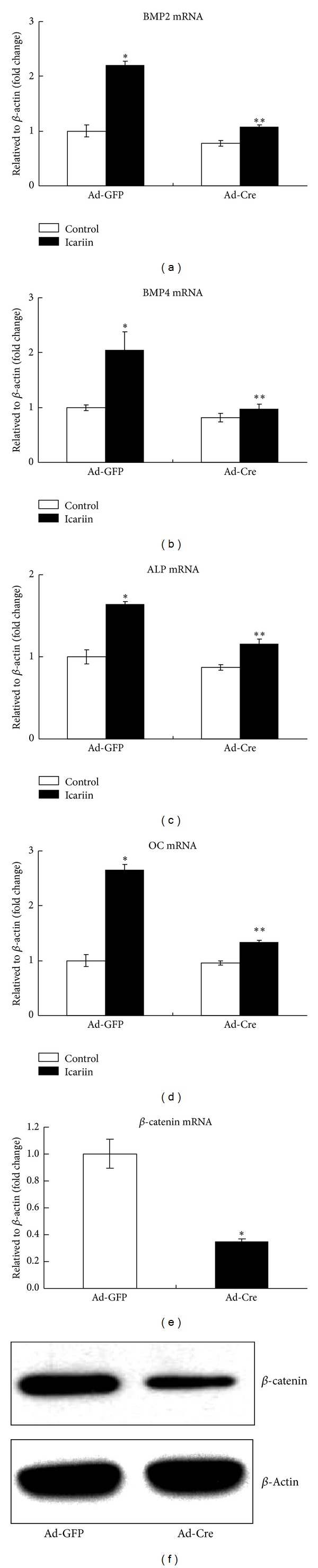
Icariin-induced osteoblast differentiation in a *β*-catenin-dependent manner. Ad-Cre infection significantly inhibited Icariin-induced expression of BMP2 (a), BMP4 (b), ALP (c), and OC (d). Ad-Cre-mediated deletion of the *β*-catenin gene was demonstrated by the reduction of *β*-catenin mRNA (e) and protein (f) expression in these cells. Data were expressed as mean ± SD, *n* = 3. *P < 0.05, unpaired Student's* t-*test (Ad-GFP + Icariin group versus Ad-GFP + Control group) and **P < 0.05, unpaired Student's* t-*test (Ad-Cre + Icariin group versus Ad-GFP + Icariin group).

To further determine if the effect of Icariin on osteoblast differentiation is *β*-catenin dependent, primary bone marrow stromal cells were isolated from femur and tibia of the *β*-catenin^fx/fx^ mice and infected with either Ad-GFP or Ad-Cre. The cells then were treated with Icariin (50 *μ*M) for additional 2 days, and the effects of Icariin on the expression of osteoblast marker genes were examined. Infection of Ad-Cre efficiently deleted the *β*-catenin gene in bone marrow stromal cells (data not shown). In Ad-GFP group, Icariin significantly up-regulated the expression of BMP2 (2.1-fold increase) (Figure 7(a)), BMP4 (2-fold increase) (Figure 7(b)), ALP (1.6-fold increase) (Figure 7(c)), and OC (2.7-fold increase) (Figure 7(d)). While, Ad-Cre infection significantly inhibited the Icariin-induced expression of BMP2 (Figure 7(a)), BMP4 (Figure 7(b)), ALP (Figure 7(c)), and OC (Figure 7(d)), Ad-Cre-mediated deletion of the *β*-catenin gene was demonstrated by the reduction of *β*-catenin mRNA (Figure 7(e)) and protein (Figure 7(f)) expression in these cells. These results suggest that Icariin induces osteoblast differentiation through activation of Wnt/*β*-catenin-BMP signaling. 

## 4. Discussion

In the present study, we found that Icariin stimulates new bone formation after local injection over the surface of calvaria. Icariin is also capable of significantly reversing OPG-deficient-induced bone loss and promotes osteoblast differentiation in OPG^−/−^ mice, which was demonstrated by the upregulation of BMP2, BMP4, RUNX2, and OC expression. The expression of Wnt1 and Wnt3a was significantly up-regulated in lumbar vertebrae of OPG mutant mice after treatment with Icariin. Treatment of Icariin in OPG^−/−^ mice increases the expression of AXIN2, DKK1, TCF1, and LEF1, which are the direct target genes of *β*-catenin signaling. We also examined the effect of Icariin on the biomechanical properties of the femoral diaphysis and found that it significantly enhances bone mechanical strength. Furthermore, in vitro studies demonstrated that Icariin induces osteoblast differentiation through the activation of Wnt/*β*-catenin-BMP signaling.

The receptor activator of NF-*κ*B (RANK) [28], its ligand RANKL [29], and the decoy receptor for RANKL, OPG [30] are the factors involved in the control of osteoclasts. Binding of RANKL to its receptor RANK provides the crucial signal to drive osteoclast development from haematopoietic progenitor cells as well as to activate mature osteoclasts. OPG negatively regulates RANKL binding to RANK and therefore inhibits bone turnover by osteoclasts. OPG gene knockout mice (OPG^−/−^) develop severe osteoporosis with remarkably increased osteoclast numbers as adults [14]. In this study, bone loss and bone strength reduction were observed in OPG^−/−^ mice at 5-month-old age. We examined the effects of Icariin on the structural and biomechanical properties of the bone in OPG^−/−^ mice, and our data showed that Icariin significantly increases bone mass, increasing trabecular bone volume and trabecular number, and enhances bone mechanical strength, demonstrated by increases in maximal force, bone stiffness, and bone energy. Although it has been reported that the beneficial effects of Icariin on bone are diminished in OPG^−/−^ mice, the extent of reduction is not large [22]. As we know that bone resorption is dominant in OPG^−/−^ mice, therefore, Icariin-induced bone formation is mainly attributable to increased bone mass and bone strength in OPG^−/−^ mice. 

Bone morphogenetic proteins (BMPs) are strong inducers of osteoblast differentiation and bone formation [31]. In this study, BMP2 and BMP4 were upregulated in OPG^−/−^ mice after the treatment of Icariin. BMP2 acts on bone cells by binding to their cell surface receptors and subsequently phosphorylating Smads1/5/8. Phosphorylated Smads1/5/8 forms a complex with Smad4 and translocates to the nucleus, whereby it regulates the transcription of bone-specific genes [32]. Several lines of evidence have demonstrated that BMP2 induces or promotes the expression of RUNX2 and Osterix [33], which are essential transcription factors for osteoblast differentiation and bone formation [34], as well as osteoblast differentiation markers such as alkaline phosphatase (ALP), type I collagen (COL1), and osteocalcin (OC) in various cells. Polymorphisms in the BMP4 gene are also demonstrated to be associated with senile osteoporosis. BMP2 and BMP4 have stimulatory effect on the expression of COL1, ALP, and OC [25, 34]. Thus, BMPs are key molecules in the regulation of osteoblast differentiation. 

Our recent studies demonstrated that BMP expression is activated by canonical Wnt/*β*-catenin signaling in osteoblast cells and in bone marrow stromal cells [26, 27]. Zhang et al. also reported that Wnt/*β*-catenin signaling activates bone morphogenetic protein 2 expression in osteoblasts through a TCF-*β*-catenin enhancer [35]. To determine the mechanism by which Icariin promotes the expression of BMPs and stimulates osteoblast differentiation, we examined its effect on Wnt/*β*-catenin signaling. Our in vitro studies indicate that Icariin activates the canonical Wnt/*β*-catenin signaling pathway in osteoblast cells. We found that deletion of the *β*-catenin gene in osteoblasts significantly inhibits Icariin-induced osteoblast differentiation, evidenced by the inhibition of BMP2, BMP4, ALP, and OC expression. These findings suggest that Icariin may stimulate osteoblast differentiation through activation of canonical Wnt/*β*-catenin-BMP signaling pathway.

In summary, this study clearly demonstrates that Icariin can induce new bone formation, preventing loss of bone and bone strength caused by the deletion of the OPG gene. Additionally, these findings purport that such effects may be mediated by inducing the expression of BMPs, leading to osteoblast differentiation. To up-regulate BMP expression, Icariin may activate the Wnt/*β*-catenin signaling pathway. Taken together, our data indicate that Icariin increases bone mass and bone strength in OPG-deficient mice through the activation of Wnt/*β*-catenin-BMP signaling. Our future research will focus on optimizing the pharmacokinetics and pharmacodynamics of Icariin for possible oral administration.

## Figures and Tables

**Table 1:** Mouse primers for real-time quantitative PCR assays.

Genes	Forward primer	Reverse Primer
*β*-actin	TGTTACCAACTGGGACGACGACA	CTGGGTCATCTTTTCACGGT
ALP	TGACCTTCTCTCCTCCATCC	CTTCCTGGGAGTCTCATCCT
BMP2	ACTTTTCTCGTTTGTGGAGC	GAACCCAGGTGTCTCCAAGA
BMP4	GAGGAGGAGGAAGAGCAGAG	TGGGATGTTCTCCAGATGTT
RUNX2	TCCTGTAGATCCGAGCACCA	CTGCTGCTGTTGTTGCTGTT
OC	CTTGAAGACCGCCTACAAAC	GCTGCTGTGACATCCATAC
Wnt1	ACAGCGTTCATCTTCGCAATCACC	AAATCGATGTTGTCACTGCAGCCC
Wnt3a	GGCTCCTCTCGGATACCTCT	GGGCATGATCTCCACGTAGT
AXIN2	AGTCCATCTTCATTCCGCCTAGC	AAGCTGCGTCGGATACTTGCGA
DKK1	ATTCCAACGCTATCAAGAACC	CCAAGGTGCTATGATCATTACC
TCF1	CCCCGATCTCTCTGGATTTT	AAGGGGACAGGGGTAGAG
LEF1	TCATCACCTACAGCGACGAG	GAAGGTGGGGATTTCAGGAG
